# The Aliviado DSD Program: A Time-Series Study on Enhancing Family Caregiver Delirium Knowledge

**DOI:** 10.1093/geroni/igaf122.1326

**Published:** 2025-12-31

**Authors:** Shih-Yin Lin, Yong Kyung Choi, Donna Fick, Abraham Brody

**Affiliations:** New York University Rory Meyers College of Nursing, New York, New York, United States; University of Pittsburgh, Pittsburgh, Pennsylvania, United States; The Pennsylvania State University, University Park, Pennsylvania, United States; NYU Rory Meyers College of Nursing, New York, New York, United States

## Abstract

Delirium superimposed on dementia (DSD) refers to delirium occurring in individuals with existing dementia. Due to the overlapping symptoms between delirium and dementia, family caregivers play an important role in detecting DSD. They can assist in DSD detection by informing clinicians whether the cognitive disturbances in their care recipients are typical day-to-day fluctuations in dementia symptoms or indicative of something beyond the normal. However, many family caregivers are unfamiliar with DSD. To assess the impact of the Aliviado DSD Caregiving Mastery Program on caregivers’ delirium knowledge, we conducted a single-group, time-series study with data collected at baseline, 2 weeks, and 6 weeks. Delirium knowledge was assessed using the Caregiver Delirium Knowledge Questionnaire. Descriptive statistics summarized participant characteristics, while a mixed-effects regression model analyzed the delirium knowledge outcome, accounting for repeated measures across time points. A random intercept was included to account for individual baseline differences. Thirty primary caregivers participated, with 26 completing the study (retention rate: 86.7%). At 2 weeks, delirium knowledge significantly increased compared to baseline (mean difference = 3.80, p < 0.001). At 6 weeks, knowledge remained significantly higher than baseline (mean difference = 3.63, p < 0.001). The intervention effect sizes from baseline to 2 weeks and baseline to 6 weeks were large (Cohen’s d = 0.8 and 0.76, respectively). No significant difference in delirium knowledge was observed between 2 and 6 weeks, suggesting knowledge maintenance. The study findings provide preliminary evidence supporting the positive impact of the Aliviado DSD program on improving caregivers’ delirium knowledge.

